# Late-life falling and depressive symptoms associated with the risk of Parkinson’s disease: a nationwide cohort data analysis

**DOI:** 10.1186/s12877-020-01691-9

**Published:** 2020-08-10

**Authors:** Yu Jin Jung, Ryul Kim, Dallah Yoo, Kyungdo Han, Jee-Young Lee

**Affiliations:** 1 Department of Neurology, Daejeon St. Mary’s Hospital, College of Medicine, The Catholic University of Korea, Seoul, Republic of Korea; 2 Department of Neurology, Inha University Hospital, Incheon, Republic of Korea; 3 Department of Neurology, Kyung-Hee University Hospital, College of Medicine, Kyung-Hee University, Seoul, Republic of Korea; 4 Department of Statistics and Actuarial Science, Soongsil University, Seoul, Republic of Korea; 5 Department of Neurology, Seoul National University-Seoul Metropolitan Government Boramae Medical Center, Seoul National University College of Medicine, 20 Boramae−ro 5−gil, Dongjak−gu, Seoul, 07061 South Korea

**Keywords:** Falling, Depression, Parkinson’s disease

## Abstract

**Background:**

This study aimed to evaluate the relationship between the history of late-life falling and the development of Parkinson’s disease (PD) and investigate whether depressive symptoms interact with falling to increase PD risk.

**Methods:**

We identified 1,223,726 subjects without PD who underwent the National Screening Program for Transitional Age at 66 years between 2009 and 2013 using the National Health Cohort database. In this program, every participant was assessed whether they experienced falling for the past six months. Depressive symptoms were evaluated with a three-item questionnaire extracted from the Geriatric Depression Scale. Incident PD was traced for a mean 4.23 ± 1.50 years. Cox proportional hazard regression models were used to assess the risk of PD by falling history with and without depressive symptoms after adjusting for other confounding variables.

**Results:**

In this cohort, the PD incidence rate was 1.30 and 1.03 cases per 1000 person-years in groups with and without falling and 1.34 and 1.00 cases per 1000 person-years in groups with and without depressive symptoms. The predictive risk of PD was increased by either a history of falling (HR = 1.24; 95% CI 1.11–1.40) or the presence of depressive symptoms (HR = 1.31; 95% CI 1.21–1.42) after adjusting for possible confounding variables. For individuals with both falling and depressive symptoms, PD risk increased further (HR = 1.66; 95% CI 1.40–1.97), but with sex-differences. The two factors increased PD risk in a sub-additive manner in men, whereas synergistically in women.

**Conclusions:**

This national cohort database shows that late-life depressive symptoms accompanied by a falling event pose an increase in the risk of PD in older adults.

## Background

Parkinson’s disease (PD) is one of the most common neurodegenerative disorders, and its prevalence is markedly increasing [1]. Given the growing recognition that neurodegeneration begins decades before the clinical appearance of parkinsonian motor symptoms, it becomes an important topic to identify early-stage or preclinical PD [2]. The proposed clinical markers of prodromal PD include hyposmia, constipation, sleep disturbances, and mood disorders [2].

Falling is a multifactorial phenomenon and may be affected by both motor and non-motor symptoms in PD patients. Factors that increase the risk of falling in PD include a compromised gait and balance relating to parkinsonism and cognitive impairments and mood disturbances, such as depression and anxiety [3]. Depression is a common non-motor symptom of PD [4] and it frequently precedes the onset of parkinsonism in PD [5].

Falls and depressive symptoms are common problems in community-dwelling older adults and are asignificant burden to society [6]. Individuals with falls and depressive symptoms share several risk factors, including a slow walking speed and reaction time, poor balance, reduced muscle strength, and cognitive impairment [7, 8]. Depressive symptoms and treatment of depression with antidepressants are associated with higher falling risk in older adults, but the relationship between depressive symptoms and falls was independent of antidepressant use and physical and cognitive factors [9]. Although depressive symptomatology was found to be consistently associated with falls in the general aged population, there have been only a few studies on the relationship between falls and depressive symptoms in PD development [10].

This study aimed to evaluate how falling events in the aged population could predict the future development of PD and investigate whether depression interacts with falling to increase PD risk in older adults. We used a large population-based cohort dataset from the special health screening program called the National Screening Program for Transitional Ages (NSPTA) provided by the Korean National Health Insurance (KNHI) service.

## Methods

### Data source and study population

The KNHI is a mandatory public health insurance system in South Korea [11], which provides a special health screening program, called the NSPTA, for people who have reached two target ages, 40- and 66 years-old [12]. In the program for the 66-year-old age group, the NSPTA includes a questionnaire designed to screen for mental health conditions and geriatric physical function [12].

We used the National Health Insurance Service-National Health Screening Cohort (NHIS-HEALS) database. The NHIS-HEALS contains demographic information such as age and sex, insurance premium (a proxy for economic status), disability status (recorded on six grades by the National Registration for Disability), the biennial National Health Screening Program (NHSP) [13] data, and information on medical service use for each individual with prescription data both at the outpatient clinic and during hospitalization under diagnostic codes following the modified version of the tenth edition of the International Classification of Diseases (ICD-10) [14]. The Korean government has implemented a benefit expansion policy for patients with PD, and this policy has compulsory registration with the governmental system at the first clinical visit with a PD diagnosis. Therefore, we identified patients with PD if individuals were both matched for the ICD-10 code of PD (G20) and registration to the Korean government’s benefit expansion policy. We considered the date of the earliest claim with a PD diagnosis as the index date. Within the NHIS-HEALS database, we identified 1,223,726 eligible individuals who had participated both in the NHSP and the NSPTA at age 66, between 2009 and 2013.

**Fig. 1:**
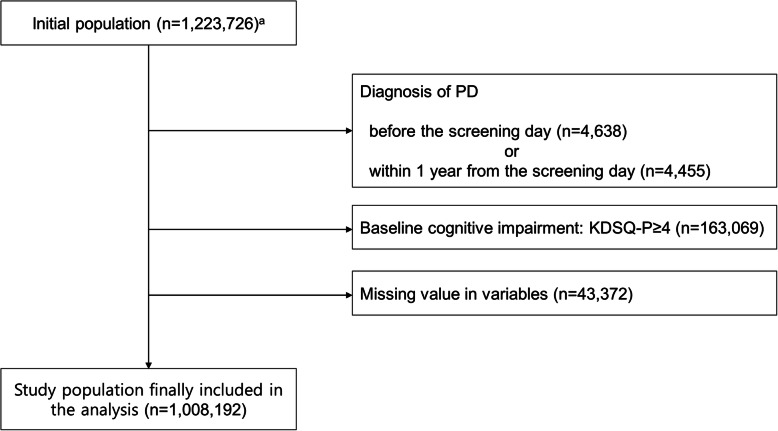
Flowchart of the study population. Among the initial 1,223,726 individuals eligible for this study, 1,008,192 subjects were finally included in the analysis by the exclusion criteria of this study. ^a^Individuals who had participated in both the National Health Screening Program (NHSP) and the National Screening Program for Transitional Ages (NSPTA) at age 66 during 2009–2013. Abbreviations: PD, Parkinson’s disease; KDSQ-P, Prescreening Korean Dementia Screening Questionnaire

We excluded individuals who had a diagnosis of PD before (*n =* 4638) and during the 1-year lag period (*n =* 4455) of the health screening date. Moreover, individuals who had baseline cognitive impairment, assessed by the scores of the Prescreening Korean Dementia Screening Questionnaire (KDSQ-P) [15] ≥4, (*n* = 163,069) were excluded, because cognitive impairment was known to be a common risk factor of falls and depressive symptoms [7, 8]. Individuals with missing data for at least one variable (*n =* 43,372) were also excluded. The final study population was followed until the occurrence of PD, death, or the last follow-up date, December 31, 2017 (Fig. 1).

### Variables

At the time of the NSPTA exam, all participants were asked whether they experienced any falling during the past 6 months. We collected information on the following variables: sex, smoking, alcohol consumption, exercise, economic status, body mass index (BMI), and comorbid conditions (hypertension, diabetes mellitus, and dyslipidemia), which have been considered to be related to the development of PD. Regular exercise was defined as ≥20 min of vigorous-intensity physical activity, three or more days a week, or ≥ 30 min of moderate-intensity physical activity, five or more days a week. To screen for depressive symptoms, three questions extracted from the Geriatric Depression Scale (GDS), including loss of interest, feelings of uselessness, and feeling without hope, were used during NSPTA. If individuals answered affirmatively to any of the three questions, they were defined as having depressive symptoms in previous studies [16, 17].

### Statistical analysis

Data are summarized as numbers with percentages for categorical variables and mean values with standard deviations (SD) for continuous variables. We analyzed baseline characteristics with student t-tests or chi-square tests. *P*-values represent statistical differences between the two groups. We used the Kaplan–Meier curve to describe the cumulative incidence of PD by time graphically and tested a statistical significance of the curves by falling or depression using a log-rank test. We also conducted multivariate analyses using a Cox proportional hazard regression model. We made adjustments for sex in model 1 and sex, smoking, alcohol consumption, exercise, and household income status in model 2. Besides, hypertension, diabetes mellitus, and hypercholesterolemia were further adjusted in model 3. The hazard ratios (HRs) and 95% confidence intervals (CIs) for the risk of PD in each model were estimated. The HRs for the presence of falling by the presence/absence of depressive symptoms for PD development were also assessed by multivariable Cox proportional hazards regression models. All statistical analyses were performed using SAS (version 9.4; SAS Institute Inc., Cary, NC) with the significance level set at 0.05 (two-tailed).

## Results

### Baseline characteristics of the study population

Finally, a total of 1,008,192 participants were included in the analysis, as described in Fig. 1. The baseline age was 66 years old. Among the 1,008,192 subjects included in the analysis, 63,939 (6.7%) stated that they had fallen in the past 6 months. The baseline characteristics of the study population are summarized in Table 1. The percentage of women was higher in the falling group compared with the no-falling group (67.2% vs. 52.0%). Individuals with falling were more likely to be non-smokers (76.4% vs. 69.0%) and non-drinkers (77.0% vs. 71.3%) and had a lower household income (25.6% vs. 24.4%) and a higher BMI (24.5 vs. 24.3). The falling group also consisted of a lower proportion of subjects with regular exercise (41.8% vs. 46.8%). Hypertension (55.9% vs. 54.2%), diabetes mellitus (22.4% vs. 20.1%), and hypercholesterolemia (40.6% vs. 36.4%) were more frequent in the falling group than those without a falling history. Depressive symptoms were also more prevalent in the falling group than those without a falling history (30.2% vs. 11.4%).

**Table 1:** Baseline characteristics by the presence of falling history

Variables	Total (***n*** = 1,008,192)	Individuals with the fall history (***n*** = 63,939)	Individuals without the fall history (***n*** = 944,253)	***P*** value
Age, years	66.0	66.0	66.0	
Sex				< 0.001
Male	474,284 (47.0)	20,961 (32.8)	453,323 (48.0)	
Female	533,908 (53.0)	42,978 (67.2)	490,930 (52.0)	
Smoking				< 0.001
Never smoker	699,442 (69.4)	48,737 (76.4)	650,705 (69.0)	
Ex-smoker	175,003 (17.4)	8450 (13.2)	166,553 (17.7)	
Current smoker	132,666 (13.2)	6642 (10.4)	126,024 (13.4)	
Alcohol^a^				< 0.001
Non-drinker	719,182 (71.7)	48,987 (77.0)	670,195 (71.3)	
Moderate drinker	244,632 (24.3)	12,504 (19.7)	232,128 (24.7)	
Heavy drinker	39,995 (4.0)	2126 (3.3)	37,869 (4.0)	
Regular exercise^b^	467,788 (46.5)	26,687 (41.8)	441,101 (46.8)	< 0.001
Household income status percentiles				0.001
≤ 20 (low)	246,929 (24.5)	16,388 (25.6)	230,541 (24.4)	
30–50	236,518 (23.5)	14,454 (22.6)	222,064 (23.5)	
60–80	317,183 (31.5)	20,073 (31.4)	297,110 (31.5)	
≥ 90 (high)	207,562 (20.5)	13,024 (20.4)	194,538 (20.6)	
Body mass index, kg/m^2^	24.32 ± 3.03	24.53 ± 3.18	24.31 ± 3.02	< 0.001
Hypertension	546,896 (54.3)	35,698 (55.9)	511,198 (54.2)	< 0.001
Diabetes mellitus	204,168 (20.3)	14,329 (22.4)	189,839 (20.1)	< 0.001
Hypercholesterolemia	369,783 (36.7)	25,918 (40.6)	343,865 (36.4)	< 0.001
Depressive symptom				< 0.001
No	879,380 (87.4)	44,523 (69.8)	834,857 (88.6)	
Yes	126,990 (12.6)	19,297 (30.2)	107,693 (11.4)	
Follow up duration	4.23 ± 1.5	4.24 ± 1.5	4.23 ± 1.5	0.147

Data are n (%) and the mean ± standard deviation. P-values are calculated by the student t-test for continuous variables and by the chi-square test for categorical variables

^a^Daily amount of alcohol consumption was categorized as follows: heavy drinker, ≥30 g/day; moderate drinker, 1–30 g/day; and nondrinker

^b^Regular exercise indicates ≥20 min of vigorous-intensity physical activity ≥3 days a week or ≥ 30 min of moderate-intensity physical activity ≥5 days a week

### Parkinson’s disease risk predicted by falling and depressive symptoms in late life

**Fig. 2:**
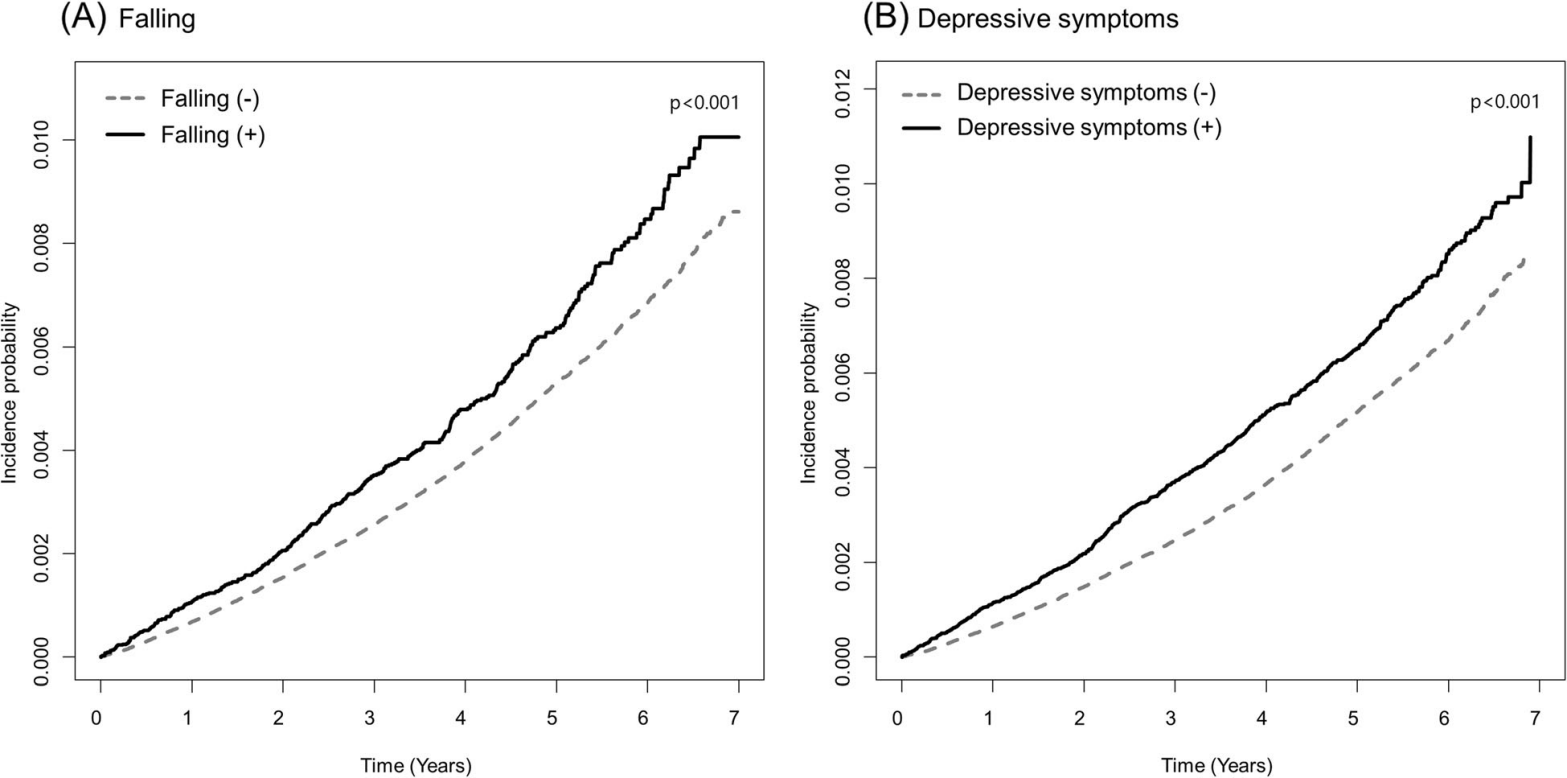
Kaplan-Meier curves for the development of Parkinson’s disease by the baseline history of falling (**a**) and depressive symptoms (**b**). The incidence of Parkinson’s disease was significantly affected by the presence of the baseline history of falling (**a**) and depressive symptoms (**b**) during the follow-up. *P*-values analyzed by log rank tests

In this study cohort, the mean follow-up period was 4.23 ± 1.5 years. The mean time to PD development was 4.23 ± 1.5 years in the falling group, whereas it was 4.24 ± 1.5 years in those without a history of falling. The incidence rates of PD were 1.30 and 1.03 cases per 1000 person-years in groups with and without falling history, respectively. Figure 2-A depicts Kaplan-Meier curves showing the relationship between the history of falling and PD incidence. PD risk was higher in the group with a history of falling than those without any falling. Unadjusted HR for PD development was 1.26 (95% CI = 1.13–1.41) in the falling group with reference to the non-falling group. In the adjusted models, a history of falling was also significantly associated with an increased risk of PD: adjusted HR = 1.25; 95% CI 1.12–1.40 in model 1, adjusted HR = 1.25; 95% CI 1.12–1.40 in model 2, and adjusted HR = 1.24; 95% CI 1.11–1.40 in model 3 (Table 2). Regarding depressive symptoms in this cohort, incidence rates of PD were 1.34 and 1.00 cases per 1000 person-years in groups with and without depressive symptoms. Depressive symptoms were significantly associated with an increased risk of PD: adjusted HR = 1.31; 95% CI 1.21–1.42 in model 1, adjusted HR = 1.32; 95% CI 1.22–1.43 in model 2, and adjusted HR = 1.31; 95% CI 1.21–1.42 in model 3 (Table 2). Figure 2-B shows the Kaplan-Meier curves for the relationship between depressive symptoms and PD incidence.

**Table 2:** Incidence of PD according to the baseline fall history and depressive symptoms

	Total number	Number of PD occurrence	Person-years	Incidence Rate^a^ (1/1000)	Adjusted HR^b^ (95% CI)		
					Model 1	Model 2	Model 3
**Individuals without the fall history**	944,253	4131	3,993,223.44	1.03	1.00	1.00	1.00
**Individuals with the fall history**	63,939	352	270,964.24	1.30	1.25 (1.12–1.40)	1.25 (1.12–1.40)	1.24 (1.11–1.38)
**Individuals without depressive symptoms**	879,380	3735	3,703,887.61	1.00			
**Individuals with depressive symptoms**	126,990	736	549,052.95	1.34	1.31 (1.21–1.42)	1.32 (1.22–1.43)	1.31 (1.21–1.42)

^a^Incidence rates were expressed as per 1000 person-years

^b^HRs were adjusted for sex (model 1), for sex, smoking, alcohol consumption, regular exercise, household income status (model 2) and for the same variables plus body mass index and the presence of hypertension, diabetes mellitus and hypercholesterolemia (model 3)

*Abbreviations*: *C*I confidence interval, *HR* hazard ratio

### Interactive impact of falling and depressive symptoms on the risk of Parkinson’s disease

**Fig. 3:**
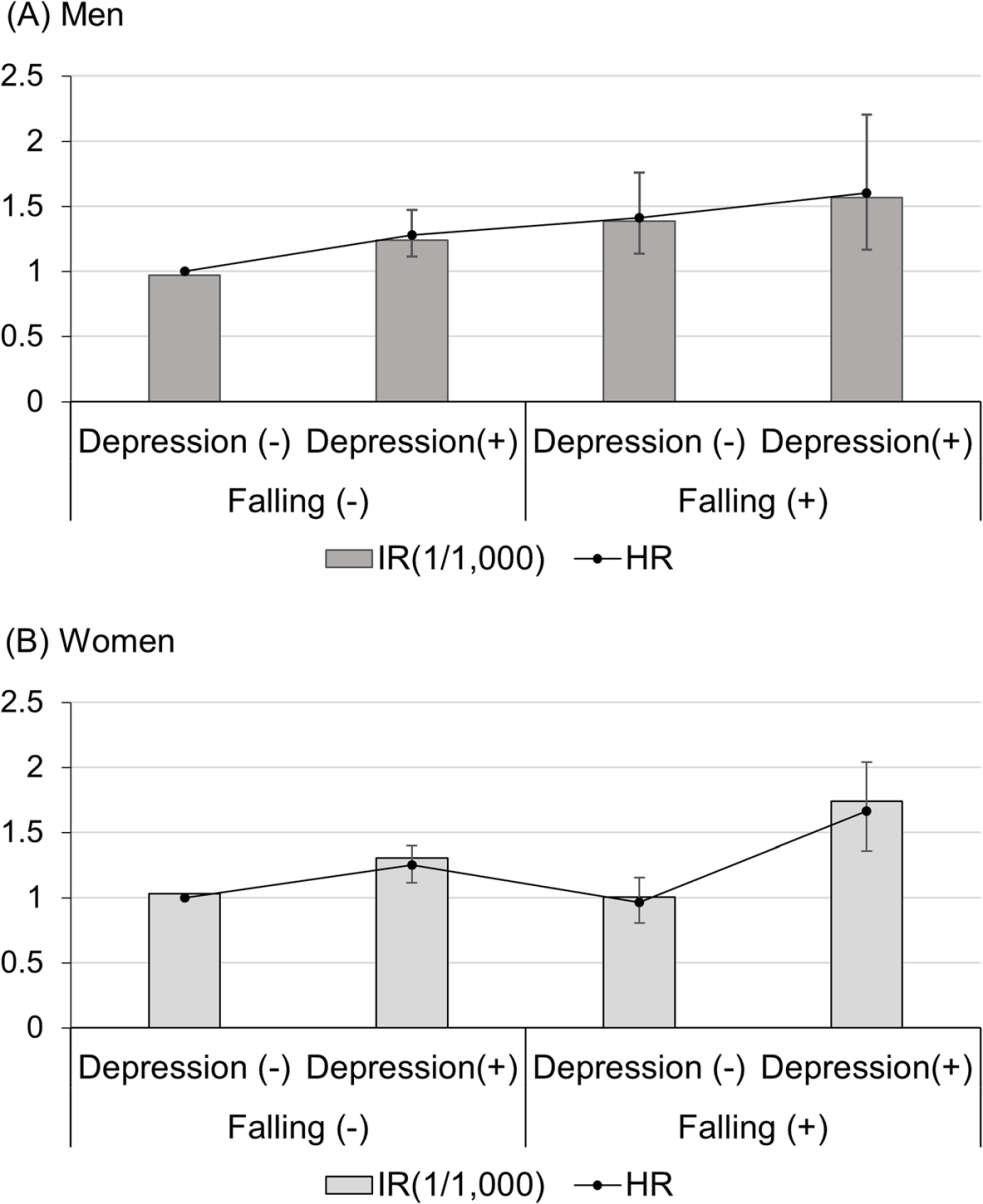
The risk of Parkinson’s disease predicted by the presence of both depressive symptoms and a history of falling at baseline in men (**a**) and women (**b**). In men (**a**), depressive symptoms and a history of falling increased the risk of PD in a sub-additive manner whereas in women (**b**), the risk of PD was synergistically increased by the presence of both a history of falling and depressive symptoms. Abbreviations: IR, incidence rate; HR, hazard ratio

As summarized in Table 3, falling in the presence of depressive symptoms seemed likely to increase the PD risk synergistically in the whole study population (HR = 1.66; 95% CI 1.40–1.97). However, when we analyzed both sexes separately, the impacts of falling and depressive symptoms were different. In men, the risk of PD increased even if either depressive symptoms or history of a falling was present (HR = 1.28; 95% CI 1.11–1.47, HR = 1.41; 95% CI 1.14–1.76) and increased further when both factors coexisted (HR = 1.60; 95% CI 1.17–2.20) in a sub-additive manner. On the other hand, in women, a history of falling alone did not increase the risk of PD (HR = 0.97; 95% CI 0.81–1.16), whereas depressive symptoms alone increased the PD risk (HR = 1.25; 95% CI 1.12–1.40). The risk of PD increased by 67% in the presence of both a history of falling and depressive symptoms (HR = 1.67; 95% CI 1.36–2.04) compared to those with double negative symptoms. Depressive symptoms with a falling history synergistically increased the risk of PD in women. (Table 3, Fig. 3).

**Table 3:** PD risk predicted by the combination of falling history and depressive symptoms

	Total number	Number of PD occurrence	Person-years	Incidence Rate^a^ (1/1000)	Adjusted HR^b^ (95% CI)
**Total**					
**Falling (−) Depression (−)**	834,857	3522	3,515,446.76	1.002	1.00
**Falling (−) Depression (+)**	107,693	598	467,281.91	1.280	1.27 (1.16–1.38)
**Falling (+) Depression (−)**	44,523	213	188,440.86	1.130	1.11 (0.97–1.28)
**Falling (+) Depression (+)**	19,297	138	81,771.04	1.688	1.66 (1.40–1.97)
**Men**					
**Falling (−) Depression (−)**	409,661	1657	1,706,433.44	0.971	1.00
**Falling (−) Depression (+)**	42,743	227	183,166.81	1.239	1.28 (1.11–1.47)
**Falling (+) Depression (−)**	14,777	86	62,030.75	1.386	1.41 (1.14–1.76)
**Falling (+) Depression (+)**	6136	40	25,498.32	1.569	1.60 (1.17–2.20)
**Women**					
**Falling (−) Depression (−)**	425,196	1865	1,809,013.32	1.031	1.00
**Falling (−) Depression (+)**	64,950	371	284,115.10	1.306	1.25 (1.12–1.40)
**Falling (+) Depression (−)**	29,746	127	126,410.11	1.005	0.97 (0.81–1.16)
**Falling (+) Depression (+)**	13,161	98	56,272.72	1.742	1.67 (1.36–2.04)

^a^Incidence rates were expressed as per 1000 person-years.^b^HRs were adjusted for sex, smoking, alcohol consumption, regular exercise, household income status, body mass index and the presence of hypertension, diabetes mellitus and hypercholesterolemia

*Abbreviations*: *CI* confidence interval, *HR* hazard ratio

## Discussion

This national cohort data analysis shows that recent falling accompanied by depressive symptoms in the aged population is associated with an increased risk of PD by 66%. One nationwide cohort study reported that 18% of PD patients, compared with 11.5% of controls, had experienced injurious falls in the 20 years before the diagnosis of PD [18] and a recent meta-analysis found that depression was associated with a 2.2-fold increase in the incidence of PD [19]. However, to the best of our knowledge, this is the first study revealing the impact of falling in combination with depressive symptoms in older adults’ future PD development.

The present study demonstrated an appreciably lower percentage (6.7%) of falls in the past six months than that (27%) of the twelve-month prevalence of falls in a recent meta-analysis, and the falling was even more prevalent in older ages [20]. The estimated pooled prevalence of depression was 34.4% in India from a meta-analysis [21], whereas our findings revealed a relatively lower incidence (12.6%) of depressive symptoms in our population. The discrepancies appeared to be due to the difference in the age and characteristics of the included subjects. Since this study was conducted on relatively young senior citizens of a single age of 66 years and based on the health screening program, the data might be more likely to reflect a healthy and active aged population than other studies.

Falling and depressive symptoms may be a sign of disconnection in the fronto-striato-limbic circuit that can be a preclinical functional change in the brain related to PD. Depression is part of the ‘emotional status’ and the amygdala is an essential neural structure of the limbic network that plays an integral role in emotional processing [22]. The amygdala and associated limbic structures have extensive efferent connections to areas involved in posture, including vestibular nuclei, the reticular formation and nuclei within the basal ganglia and nucleus accumbens [23]. Because the amygdala is one of the structures with the earliest pathological involvement of PD [24], the disruption of these amygdala-related connections in depressed older adults can hypothetically cause falling before the appearance of parkinsonian motor symptoms. Furthermore, there has been increasing evidence of a relationship between limbic dysfunction and sudden gait ignition failure or freezing in PD [25]. A functional MRI study has shown that dysfunctional fronto-striato-limbic circuitry involving emotional processing could be related to gait freezing episodes in PD patients [26]. The close association between the emotional components and disrupted gait and balance supports our finding that the combination of falling and depressive symptoms is associated with an increased risk of PD development in older adults.

The concept of “Fear of falling (FOF)” could be suggested as one possible mechanism for the interactive impact of falling and depression in the aged population. Previous falls usually play a role in developing FOF [27]. However, FOF is also commonly reported in older adults who have not experienced a real fall [28], and depression is highly associated with FOF [29, 30]. In PD, depression is a significant non-motor contributor of FOF independent of the history of real fall [10]. Therefore, depression, FOF, and falling are considerably interconnected, and they may be a sign of an increased risk of PD although the causal relationship linking depression, FOF, and the resultant falling has been unrevealed. In our cohort database, the percentage of women was higher in the falling group, and a history of falling alone did not increase the PD development, whereas the combination of falling and depressive symptoms increased PD risk in women even higher than in men. These results were in line with several other studies that the prevalence of FOF was higher in women than in men [31] and women with apathy experienced more fall incidents than men with apathy [32], thereby suggesting the possibility of “FOF” as a candidate risk factor regardless of the history of falling as predicting the risk of PD in women, which is worth to be investigated by future studies.

Our study’s strengths include the use of a homogenous sample of the Korean aged population at the same age (66 years old) and a significant follow-up period. In addition, we adjusted for potential confounders extensively in the multivariate analysis. Nevertheless, the present study has some limitations. Because this was a big data analysis, we could only track the onset time of PD by medical records, and there may be a discrepancy in the actual onset time of PD. However, we excluded individuals who had a diagnosis of PD during the 1-year lag period from the date of health screening to rule out the case in which PD has already developed. The results did not significantly vary when we analyzed the data even without the 1-year lag period. Therefore, it is less likely that our data were affected by misdiagnoses. Second, we could not include some potential confounders for PD risk in multivariate analyses. These include physical performance status, genetic causes, sleep problems and caffeine consumption. Moreover, there was a lack of information about comorbidities except for hypertension, diabetes mellitus and hypercholesterolemia and medications, which could directly affect the falling and the depressive symptoms. However, this study used nationwide big data to have enough power to avoid possible risk factor stratification. Finally, assessment of falling and depressive symptoms based on a self-reported questionnaire might be affected by recall bias.

## Conclusion

A history of recent falls accompanied by depressive symptoms is associated with a substantial increase in the risk of future PD development. The two factors increased PD risk in a sub-additive manner in men, whereas synergistically in women. Older adults who have both depressive symptoms and a history of recent falls may require a careful follow-up.

## Data Availability

Data is available from the Korea National Health Insurance Sharing Service Institutional Data Access / Ethics Committee (https://nhiss.nhis.or.kr/bd/ay/bdaya001iv.do) for researchers who meet the criteria for access to confidential data. Researchers can apply for the National health Insurance data sharing service upon approval by the Institutional Review Board of their institution. After a review by the Korea National Health Insurance Sharing Service Institutional Data Access / Ethics Committee, authors are required to pay a data access fee and confirm that other researchers will be able to access the data in the same manner as the authors.

## Abbreviations

PD: Parkinson’s disease
NSPTA: National Screening Program for Transitional Ages
KNHI: Korean National Health Insurance
NHIS-HEALS: National Health Insurance Service-National Health Screening Cohort
NHSP: National Health Screening Program
ICD: International Classification of Diseases
KDSQ-P: Prescreening Korean Dementia Screening Questionnaire
BMI: body mass index
GDS: Geriatric Depression Scale
FOF: Fear of falling
